# High-resolution estimates of tuberculosis incidence among non-U.S.-born persons residing in the United States, 2000–2016

**DOI:** 10.1016/j.epidem.2020.100419

**Published:** 2020-11-10

**Authors:** Andrew N. Hill, Ted Cohen, Joshua A. Salomon, Nicolas A. Menzies

**Affiliations:** a Division of Tuberculosis Elimination, U.S. Centers for Disease Control and Prevention, National Center for HIV/AIDS, Viral Hepatitis, STD and TB Prevention, Atlanta, GA 30329, USA; b Department of Epidemiology of Microbial Diseases, Yale School of Public Health, New Haven, CT, USA; c Department of Medicine, Stanford University, Stanford, CA, USA; d Department of Global Health and Population, Harvard T.H. Chan School of Public Health, Boston, MA, USA

**Keywords:** Tuberculosis, Generalized additive models, Incidence trends

## Abstract

In the United States, new tuberculosis cases are increasingly concentrated within non-native-born populations. We estimated trends and differences in tuberculosis incidence rates for the non-U.S.-born population, at a resolution unobtainable from raw data.

We obtained non-U.S.-born tuberculosis case reports for 2000–2016 from the National Tuberculosis Surveillance System, and population data from the American Community Survey and 2000 U.S. Census. We constructed generalized additive regression models to estimate incidence rates in terms of birth country, entry year, age at entry, and number of years since entry into the United States and described how these factors contribute to overall tuberculosis risk.

Controlling for other factors, tuberculosis incidence rates were lower for more recent immigration cohorts, with an incidence risk ratio (IRR) of 10.2 (95 % confidence interval 7.0, 14.7) for the 1950 entry cohort compared to its 2016 counterpart. Greater years since entry and younger age at entry were associated with substantially lower incidence rates. IRRs for birth country varied between 8.86 (6.78, 11.52) for Somalia and 0.02 (0.01, 0.03) for Canada, compared to all non-U.S.-born residents in 2016. IRRs were positively correlated with WHO predicted incidence rate and negatively associated with wealth level for the birth country. Lower country wealth level was also associated with shallower declines in tuberculosis over time.

Tuberculosis risks differ by several orders of magnitude within the non-U.S.-born population. A better understanding of these differences will allow more effective targeting of tuberculosis prevention efforts. The methods presented here may also be relevant for understanding tuberculosis trends in other high-income countries.

## 1. Introduction

Most individuals infected with *Mycobacterium tuberculosis* develop a subclinical latent tuberculosis infection (LTBI), which can progress to active disease many years later (Barnett et al., 1971). In high-income, low tuberculosis (TB) burden countries in Western Europe, North America, and Australasia, cases of ‘reactivation TB’ are now the primary driver of TB incidence. Delayed progression is particularly relevant for individuals migrating from high- to low-disease prevalence settings, as high infectious exposure prior to migration can cause elevated incidence of clinical disease after arrival. This is increasingly the case for TB in higher-income countries of destination. Among 15 high-income countries in Western Europe, the percentage of TB cases occurring among non-native-born populations in 2018 ranged from 51 % in Ireland to 90 % in Sweden (European Centre for Disease Prevention and Control, 2019; International Monetary Fund, 2019). In New Zealand and Australia this percentage was 78 % (Institute of Environmental Science and Research Ltd, 2015) and 86 % (Toms et al., 2017), respectively, for 2014. In the United States, the corresponding percentage has risen from 47 % to 70 % between 2000 and 2018 (U.S. Centers for Disease Control and Prevention, 2019) and epidemiological projections suggest continued increases (Menzies et al., 2018a). Evidence from transmission linkage studies suggests that the majority of new U.S. TB cases arise from reactivation of established LTBI, rather than recent infection (Yuen et al., 2016). Recent evidence also suggests that an increasing fraction of U.S. TB cases among non-U.S.-born persons arise among older individuals and those who have been in the United States for 10 years or more (Armstrong et al., 2019; Tsang et al., 2017).

Given their major contribution to TB incidence, non-native-born individuals represent a priority group for TB prevention and enhanced case surveillance. However, in many high-income countries, non-native-born individuals represent a substantial fraction of the population and targeting all such individuals for these services would require covering a large number of people. For example, non-U.S.-born individuals living in the United States in 2017 represented 13.7 % of the population, or 44.5 million individuals (U.S. Census Bureau, 2018). More precise approaches for targeting services could allow more efficient use of scarce prevention resources and reduce potential harms for those expected to receive minimal benefit from screening and treatment to prevent TB. In this study we report the distribution of TB incidence in the non-U.S.-born population between 2000 and 2016, in order to describe trends within and across migrating cohorts and identify high-risk groups that might benefit most from prevention and enhanced surveillance. We used case notification data from the U.S. National TB Surveillance System (NTSS) (U.S. Centers for Disease Control and Prevention, 2016), and population estimates derived from the American Community Survey and 2000 Decennial Census (Ruggles et al., 2015). These data represent 122, 118 non-U.S.-born TB cases, arising during more than 620 million person-years of exposure. This cohort is substantially larger than analogous cohorts from other similar settings (Aldridge et al., 2016; McBryde and Denholm, 2012; Chemtob et al., 2015; Menzies et al., 2018b) and provides a high-resolution description of differences in TB risk within the non-U.S.-born population.

## 2. Methods

### 2.1. TB case data

From the NTSS, we obtained all TB cases reported among non-U.S.-born individuals within jurisdictions comprising the 50 states and District of Columbia over 2000–2016. ‘Non-U.S.-born’ was defined as reporting birthplace outside of the United States or U.S. territories, excluding individuals born to a U.S. citizen parent, consistent with U.S. Census definitions (US Census Bureau, 2019). Variables obtained included birth country, year the TB case was reported, current age, and number of years resident in the United States.

### 2.2. Population estimates

To derive population estimates for the non-U.S.-born population, we obtained individual-level data from the American Community Survey from 2001 to 2016, and the 5% sample of the 2000 U.S. Census (Ruggles et al., 2015). We extracted variables for birth country, year of U.S. entry, current age, and survey analysis weights. We assumed smooth changes in total population within each entry-year cohort over time to reduce sampling uncertainty, and adjusted values for misclassification and underreporting biases (Jensen et al., 2015). A detailed description of methods to calculate population denominators for estimated incidence rates is provided elsewhere (Menzies, 2019).

### 2.3. Analysis strata

We tabulated TB cases and population estimates by birth country, entry year, age at entry, and years since entry. We bottom-coded entry year at 1949, and top-coded current age at 91. ISO 3166-1 alpha-3 codes (ISO3) were used to identify countries of birth. Individuals were assigned ISO3 codes according to their reported birth country. Where reported birth country referred to a dependent territory without an ISO3 code, individuals were assigned to the governing state of the dependent territory. Countries establishing independence after 2000 (Kosovo, Montenegro, Serbia, South Sudan, Timor-Leste) were pooled with the state they were part of before independence (Yugoslavia, Sudan, Indonesia), to allow consistent definitions of birth country across the study period. We excluded individuals outside of the top 100 countries of birth according to average U.S. population over the study period, and individuals recorded as having their entry year before their birth year (4.4 % and 0.02 % of TB cases, and 1.7 % and 0.03 % of the population denominator, respectively). Two more countries were dropped as they no longer existed in 2016 (U.S.S.R., Yugoslavia). This tabulation produced 7.2 million unique strata. For TB case reports, number of years since entry was missing for 4.6 % of observations and age was missing for <0.01 %. We applied multiple imputation (Honaker et al., 2011) to these missing values while enforcing the logical consistency requirement that number of years since entry be less than an individual’s age. Entry year was calculated as the difference between the year a case was reported and number of years resident in the United States.

### 2.4. Statistical analysis

We constructed two regression models predicting incident TB cases. The first model included entry year, age at entry, and number of years since entry as predictors. The second model included these terms as well as birth country. We included indicator variables for both year of and number of years since entry, rounded to the nearest decade (for example, 1960 as entry year) to adjust for recall bias spikes in respondents’ reporting in the raw data. We used a negative binomial model with a log-link function, and with population estimates included as offsets. The effect of covariates was described using generalized additive models (Hastie and Tibshirani, 1990), which extend the structure of generalized linear models with sums of smooth functions of covariates. Smoothing was implemented via thin-plate splines (Wood, 2003), allowing considerable flexibility in fitting to multiple covariates, while simultaneously penalizing over-flexible fits. These smooth terms were specified for all continuous variables (entry year, age at entry, years since entry). Birth country was incorporated using random effects for intercept and slope. Model structure selection was guided by Akaike Information Criterion. We estimated regression models for five multiply-imputed datasets, a number compatible with the small levels of missingness, and combined results using conventional methods (Rubin, 1987) to reflect uncertainty associated with missing values. Confidence intervals were simulated by sampling regression coefficients from a multivariate normal distribution defined by the estimated coefficient vector and variance-covariance matrix, and accounting for additional variance associated with multiple imputation. Analyses were performed in R v.3.5.1 (R Core Team, 2018) using the ‘mgcv’ library v.1.8–28 (Wood, 2017).

## 3. Results

The two regression models yielded smoothed TB incidence trends among the non-U.S.-born resident population, enabling stratification by age, entry year, and length of time in the United States, and, in the case of the second model, by birth country.

### 3.1. Comparison of model fit with raw data

**Fig. 1.**
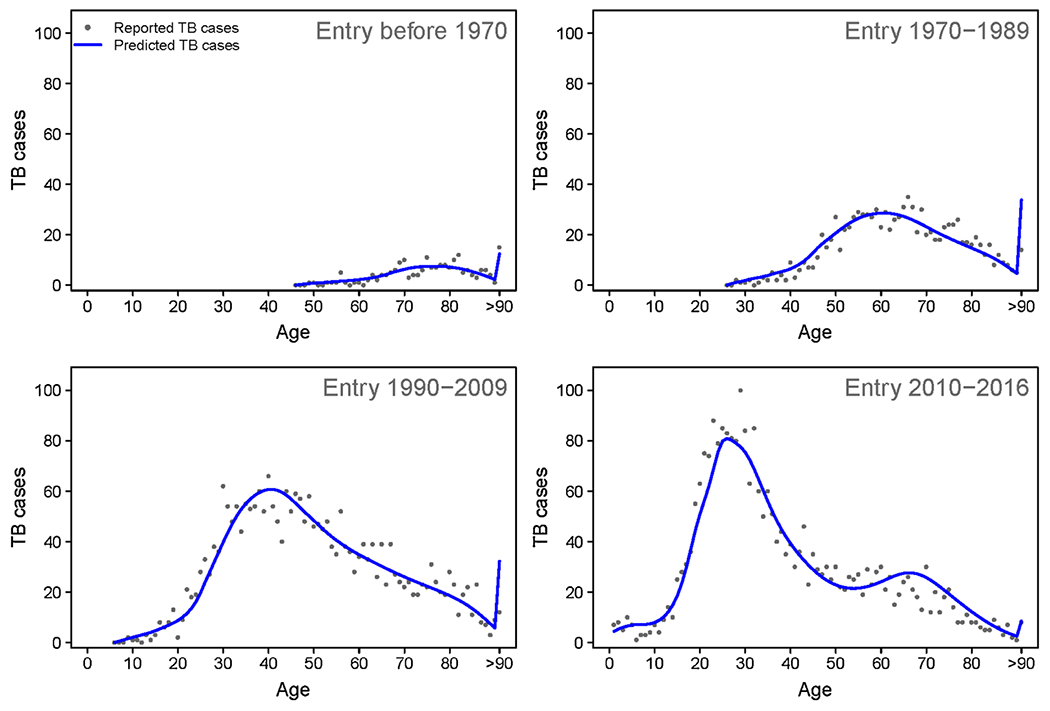
Raw and predicted non-U.S.-born TB cases for 2016 by single year of age and entry period.

Fig. 1 provides an example of raw versus smoothed estimates for total TB cases in 2016 by single year of age and stratified by epoch of entry into the United States. The model reproduces trends in the reported case counts.

### 3.2. Incidence trends for individual decade of entry cohorts

**Fig. 2.**
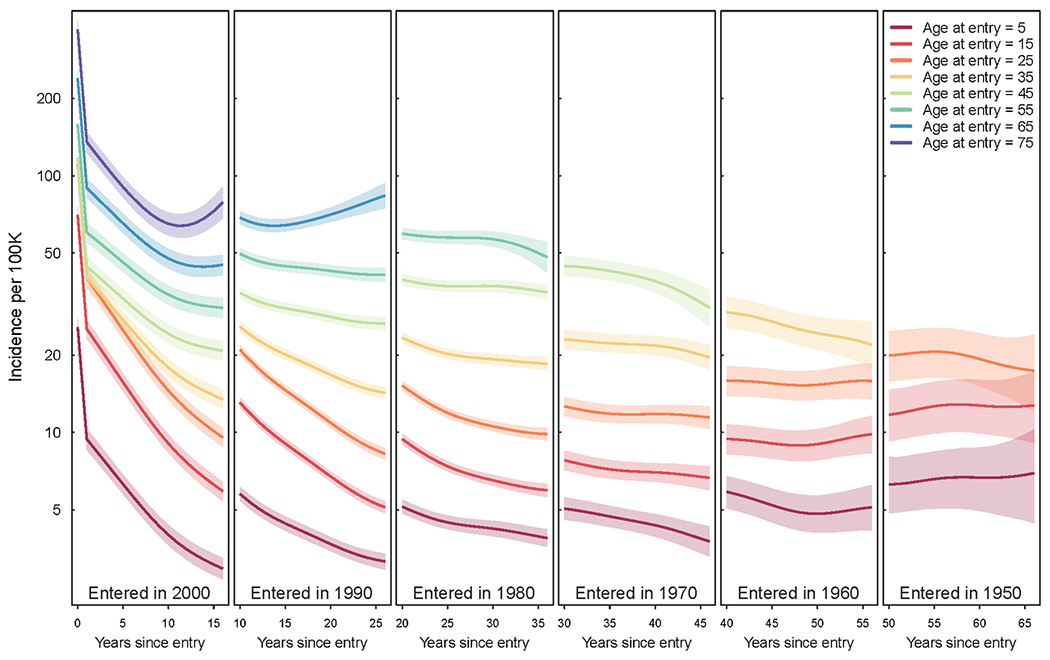
Estimated annual TB incidence per 100,000 among non-U.S.-born, by years since entry, for successive decade of entry cohorts in 2000–2016.

Fig. 2 shows estimated TB incidence trends over the period 2000–2016, stratified by 10-year age groups, for cohorts defined by their decade of entry into the United States (1950, 1960, 1970, 1980, 1990, 2000). Because each panel depicts a separate cohort, incidence estimates by age group do not connect across panels. The panels reflect the changing demographics and TB profiles over historical waves of entrants. Solid lines indicate point estimates and colored bands 95 % confidence intervals. Bands are progressively fewer moving to the right, corresponding to increasing age by the year 2000 and top-coding of age in the model. Confidence bands in the rightmost panels are wider, as expected, given fewer cases and smaller population sizes in the elderly. Incidence rates are high within the first years of arrival (leftmost panel only) and increase with age at entry. Rates generally declined within each cohort over the 17-year period, with a few exceptions: oldest age groups of the most recently arrived cohort and cohorts that arrived in 1950 and 1960. For all cohorts there was a monotonic relationship between incidence rate and age at entry, with older age at entry (cooler colors) consistently associated with higher incidence rates, compared to other individuals arriving in the same year. Across the cohorts shown in this figure, incidence rates varied from 3 to 300 per 100,000 per year.

### 3.3. Incidence predictions for cohort entering in 2016

**Fig. 3.**
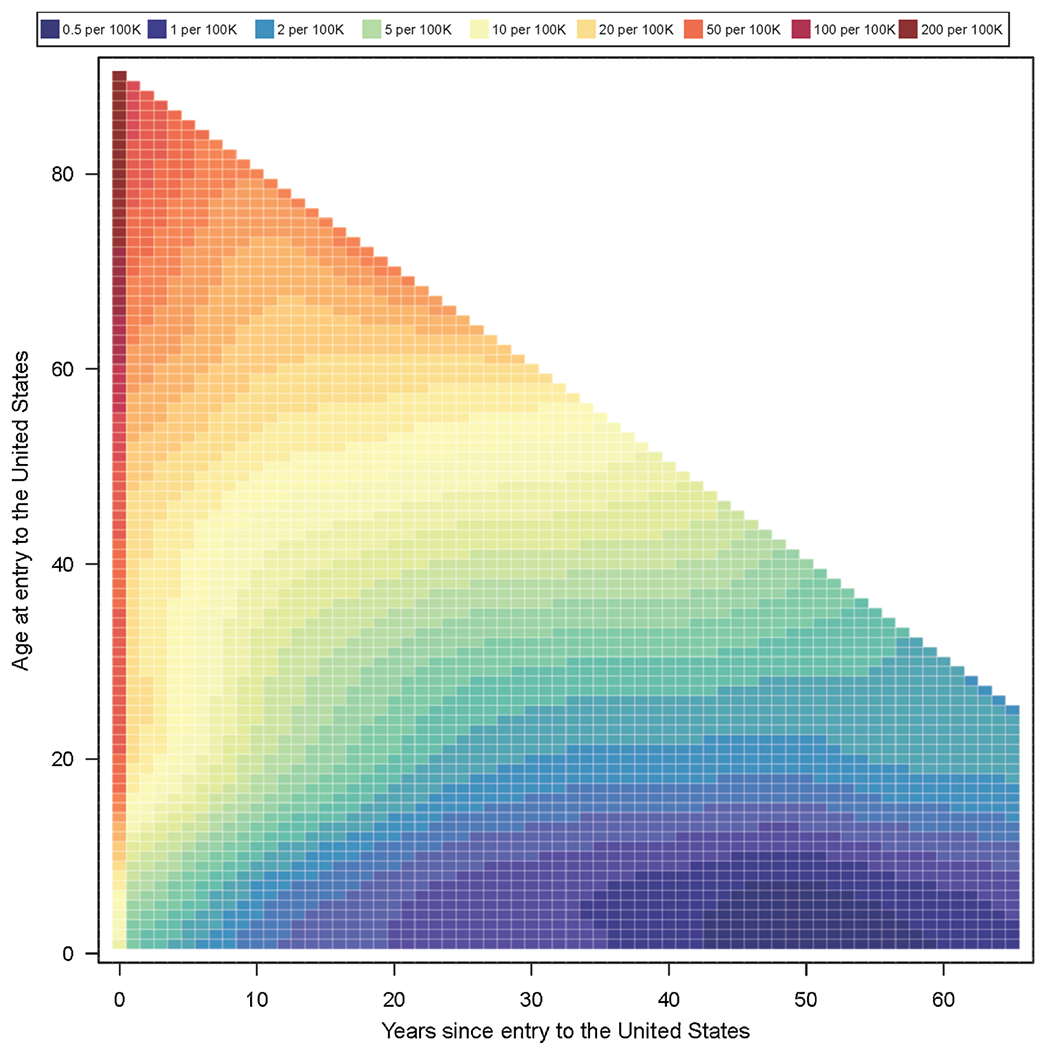
TB incidence predictions in non-U.S.-born residents by entry age and years since entry, for the 2016 entering cohort.

Fig. 3 shows a heatmap of predicted incidence rates for the 2016 entry cohort, stratified by age at entry (vertical axis) and number of years since entry (horizontal axis). Warmer colors (red) indicate higher incidence rates and cooler colors (violet) lower rates. Moving in a horizontal line from left to right traces the trajectory of predicted future incidence rates with increasing duration of U.S. residence. For all ages of entry, rates are highest immediately after entry, and absolute incidence rates are highest for individuals at advanced age when they arrive in the United States. Predicted rates decrease with time for most ages of entry, rising again in later years for those who entered as adolescents and among some who entered at older ages. As with Fig. 2, incidence rate estimates differ by two orders of magnitude across the individual strata represented in the figure.

### 3.4. Changes in incidence between successive entry year cohorts

**Fig. 4.**
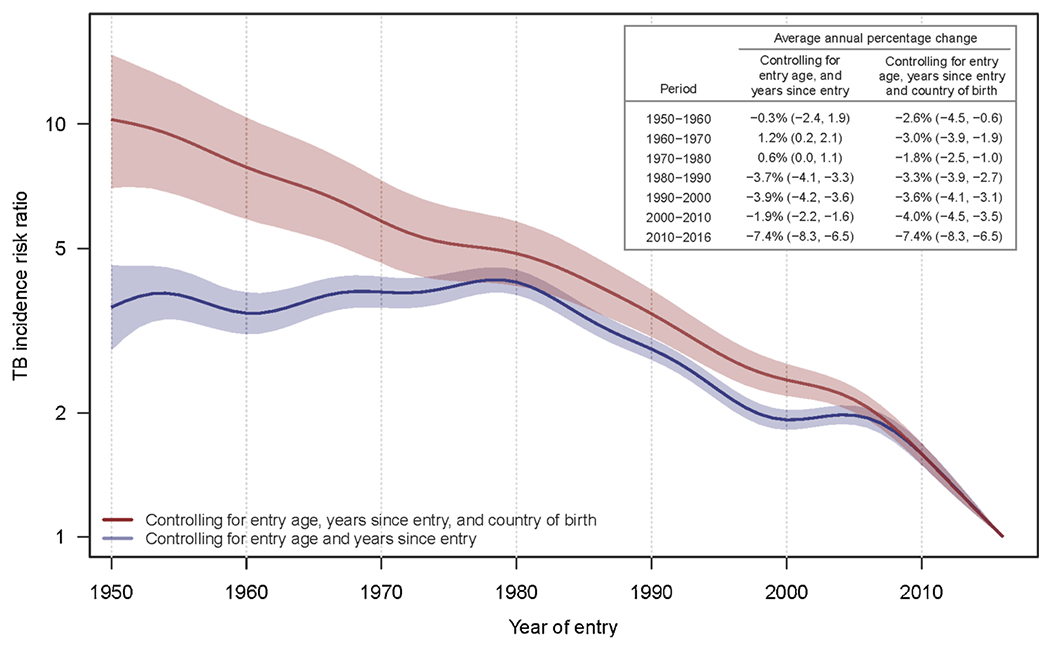
Incidence risk ratio among non-U.S.-born residents by entry year, relative to 2016 entry cohort.

Fig. 4 illustrates the secular trend in TB among successive entry cohorts between 1950 and 2016, controlling for other factors. Trends are represented by incidence risk ratios (IRRs) relative to TB morbidity in the 2016 entry cohort. The effect of controlling for birth country is apparent when comparing trends from the two regression models. For the analysis excluding birth country (blue curve), the IRR increases slightly between 1950 and 1980 (a period during which immigration shifted from predominantly European countries toward immigration from Asian and Latin American countries) and decreases thereafter. For the analysis including birth country (red curve), the IRR starts out in 1950 at 10.2 (95 % confidence interval 7.0, 14.7) relative to the rate in 2016 and decreases steadily for more recent entry years. The two curves decrease at similar rates per decade from 1980 onwards, except for 2000–2010, as shown by average annual percent changes calculated for each decade.

### 3.5. Incidence trends according to birth country

Table 1 gives raw incidence rates for persons from selected countries in 2016, as well as raw and adjusted IRRs compared to the overall non-U.S.-born rate in the same year. Results are shown for persons from the five countries with largest IRR, the five contributing most numbers of cases, and the five with smallest IRRs. The highest five IRRs range from 5.6 to 8.9; the lowest five are virtually level at 0.02 to 0.03. Supplementary Table 1 provides estimates for all birth countries.

**Fig. 5.**
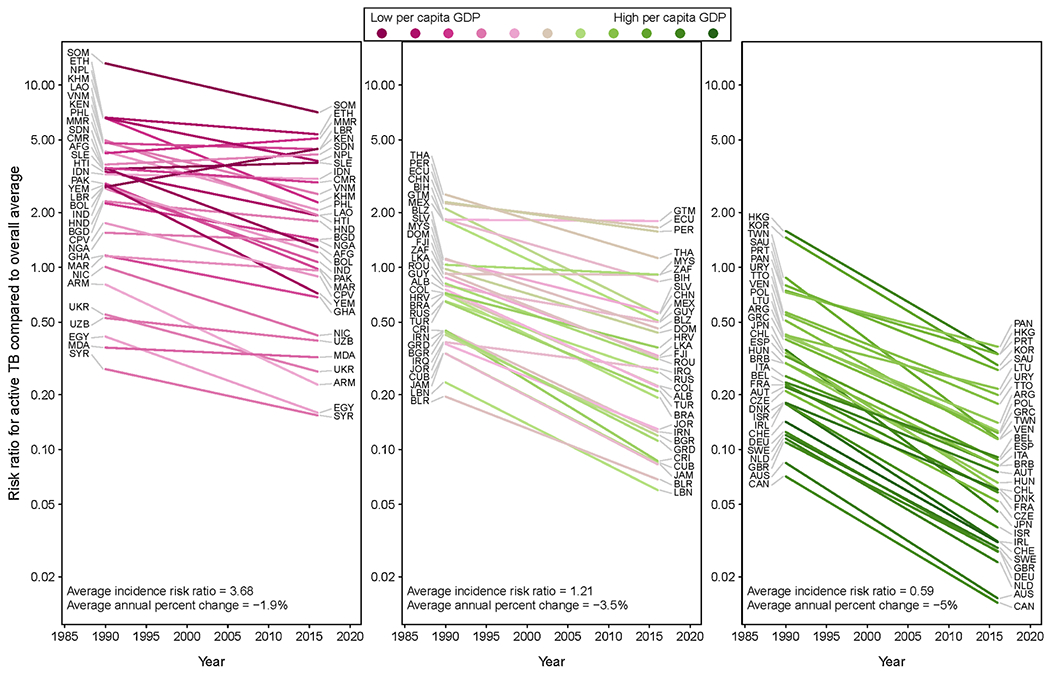
Trends in incidence by birth country and entry year for the top 98 countries-of-origin by resident population size, categorized by relative gross domestic product. Three letter codes represent country ISO3 identifiers; see Supplemental Table for list.

Fig. 5 shows time trends in TB for successive immigration cohorts, stratified by birth country. These are represented as IRRs relative to the incidence rate for all non-U.S.-born residents in 2016. IRRs decline over time for most countries, consistent with the overall patterns in Fig. 4. Countries with IRRs estimated to increase over time were Myanmar, Liberia, Sudan, Sierra Leone, Guatemala, and South Africa.

Fig. 5 also describes the relationship between wealth level in the birth country (measured as 2016 per capita GDP (The World Bank, 2018)), and TB trends for individuals from that country residing in the United States. Countries are organized in the figure by increasing per capita GDP, with countries in the lowest wealth tertile on the left, then mid-tertile countries, and the highest wealth tertile on the right. Across these panels the average 2016 IRR decreases from 3.68 for the poorest tertile to 0.59 for the wealthiest tertile. The average annual percentage change in these IRRs goes from −1.9 % in the poorest tertile to −5.0 % in the wealthiest tertile. In short, TB incidence is lower and declines more rapidly for non-U.S.-born residents from wealthier countries.

### 3.6. Relationship to TB incidence in birth country

**Fig. 6.**
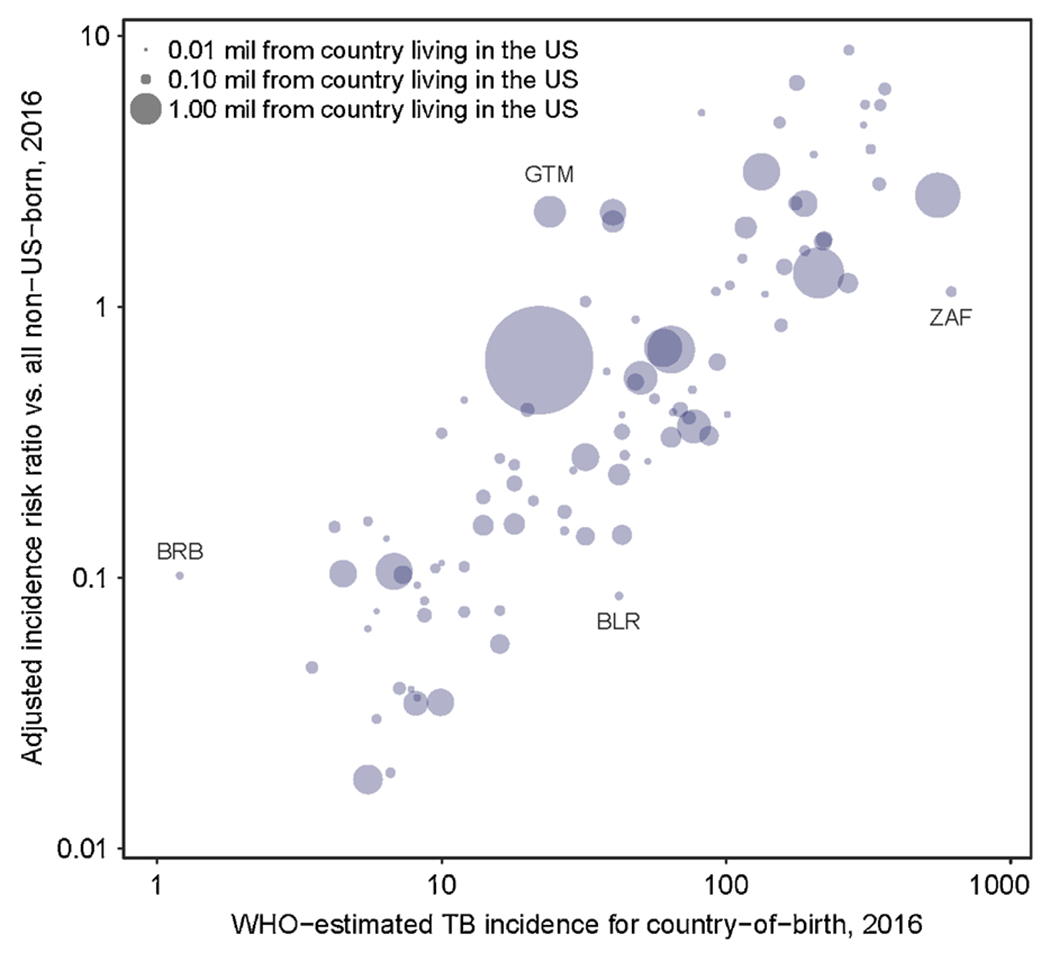
TB incidence risk ratios in 2016 relative to overall non-U.S.-born incidence compared to WHO-estimated TB incidence in birth country.

Fig. 6 compares IRR estimates relative to overall non-U.S.-born for persons from each birth country to the World Health Organization’s (WHO) TB incidence estimates for those countries in 2016 (World Health Organization, 2019). Countries are represented in the bubble plot by the size of their population in the United States. While there is a clear positive relationship between IRR and estimated TB incidence in the birth country, a number of countries deviate from this trend. Some countries with high rates of TB, such as South Africa (ZAF) have U.S. rates close to the overall non-U.S.-born population. Although Mexico (largest dot in Fig. 6) represents the birth country for about one-fifth of cases among non-U.S.-born persons, the U.S.-incidence rate among persons born in Mexico is below that of the overall non-U.S.-born population. By contrast, Guatemala (GTM), with a similar TB rate to Mexico, has rates in the United States higher than the overall average for non-U.S.-born. Noted on the edges of the scatterplot are Barbados (BRB), with an IRR much higher than would be predicted by its WHO incidence rate, and Belarus (BLR), with an IRR lower than would be predicted by its WHO incidence rate.

## 4. Discussion

In recent decades the epidemiology of TB in the United States has shifted towards reactivation of latent infection as the main driver of new cases of disease. The majority of new TB cases now arise among the non-U.S.-born population, reflecting elevated exposure to infection before U.S.-arrival. As TB becomes increasingly concentrated in the non-U.S.-born population, it is important to distinguish differences in risk within this population, to better understand the current state of the TB epidemic in the United States.

For this study we analyzed data on 122,118 non-U.S.-born TB cases arising over 620 million person-years of exposure between 2000 and 2016. This large dataset allowed us to document major differences in TB risk within the non-U.S.-born population as a function of age and entry year, years since entry, and birth country. The interaction of these factors produces incidence risks for individual population strata that differ by several orders of magnitude and suggests clear opportunities for TB prevention programs. These granular insights would not have been possible to discern directly from the raw data of case counts; a previous analysis which pooled case data over several years to address sparse counts had much more limited ability to resolve the epidemiology of TB among the non-U.S.-born (Cain et al., 2008).

Our approach allowed us to estimate the component of TB risk attributable to the year in which an individual moved to the United States. We found that earlier entry cohorts experienced a higher risk of incident TB between 2000–2016, controlling for other factors associated with TB risk. For each of these arrival cohorts non-U.S.-born rates were found to be highest in the first few years since entry and increase with age at the time of arrival. While this is a well-known feature of TB epidemiology in the non-U.S.-born, our analytic approach allowed us to project these trends over the lifetime of an individual arrival cohort, demonstrating incidence risk to continue declining up to 40 years after arrival (though at a slower rate than observed shortly after entry). Within a given entry cohort, incidence rates were found to drop quickly with years since entry for those entering at a younger age, a finding consistent with the fact that infants and young children with TB would have developed disease as a result of recent infection. By contrast, older entrants retain a higher level of risk over time since entry, reflective of higher rates of reactivation from infection in the past. Rates were projected to rise again in later years among some who entered in 2016 at older ages and this is consistent with the upturn observed among older entrants in cohorts arriving in 1990 and 2000.

Comparison of the two regression models confirmed the importance of accounting for birth country to understand differences in TB incidence rates between successive cohorts. By doing so, our model incorporates the changing demographics over time of the originating countries, where more immigrants came from Western Europe in the mid-20th century as opposed to greater proportions of arrivals from Latin America and Asia in the latter part of the 20th century through to the present day. A steady decrease of IRR relative to incidence in 2016 has mostly prevailed since 1950 for individual countries of birth and failing to control for birth country was found to mask this phenomenon.

Per capita GDP of the birth country was found to be a strong predictor of incidence rates in the United States, with the top five countries by IRR falling in the bottom third of countries with respect to per capita GDP. While the top five countries in terms of annual cases (Mexico, Philippines, India, Vietnam, China) currently receive greater attention in the United States, there remain smaller populations within the United States whose TB incidence rates are considerably higher (U.S. Centers for Disease Control and Prevention, 2019; Tsang et al., 2020). If these populations are relatively localized in the United States, these groups might benefit from additional TB prevention and control resources. It is noteworthy that Mexico and China are in the middle tertile of per capita GDP and have rates below the overall non-U.S.-born rate (IRR < 1) whereas the Philippines, India, and Vietnam are all in the lower per capita GDP tier and have rates above the average. The model also identifies birth countries for which more recent entry cohorts have higher TB incidence, controlling for age at entry and years since entry. In particular, more recent entrants from Myanmar (MMR), Liberia (LBR), and Kenya (KEN) are predicted to face greater TB risks than earlier entrants from these countries.

Our analytic approach has limitations associated with estimation of population denominators which have previously been discussed (Menzies, 2019). While NTSS data are largely complete and consistent (Yelk Woodruff et al., 2015) notwithstanding small levels of missingness noted here, data sources informing the numerator and denominator are subject to biases, for example in NTSS with recall peaks for initial or mid-years of decades and some data discontinuities in first year since entry reporting. We attempted to correct for these expected sources of bias, but our models would not capture unexpected disruptive events affecting denominators (for example, refugee inflows in certain years) or numerators (such as the unexpected drop in TB cases in 2008–09 (Winston et al., 2011)).

Model results for a specific calendar year describe only those individuals in historic entry cohorts who have survived to that year. Results should not be interpreted as referring to the complete cohort arriving in any past year. Estimates could thus be biased if there were, for example, higher survival rates among those from higher income or lower TB prevalence countries, in which instance we would be underestimating rates among historic entry cohorts.

Country-specific IRRs defined as the WHO-estimated TB incidence rate divided by the non-U.S.-born rate for that country were recently published elsewhere (Tsang et al., 2020). These IRRs should not be confused with ours which are relative to the overall non-U.S.-born rate in a given year; comparators are explicitly stated throughout the manuscript whenever IRRs are stated. An IRR > 1 here indicates elevated rates for a specific country whereas in (Tsang et al., 2020) it indicates a lower rate for the country in question (when compared to the WHO rate).

In this work we adopted an analytic approach that attempted to separate systematic trends in the data from the sampling uncertainty associated with small population strata and observed reporting biases. This led us to specify flexible functions to describe the relationship between continuous predictors and incidence rates, and random effects to regularize the estimates for individual countries of birth. Our approach required analytic decisions that might have led to both over- or underfitting of the data. As an example of the former, it is possible that the sharp increase in incidence rates 10 years after entry for individuals aged 75 years old in the 2000 entry cohort (Fig. 2, top left) results from over-fitting to random variation in the empirical data. For the latter, our decision to include different countries of birth in the same model may have hidden patterns specific to individual countries. More closely-fitted models could be developed in future work, with the usual tradeoffs in interpretability and accuracy (Caruana et al., 2015). Indeed, development of models specifically for individual countries of birth or sub-national geographies within the United States could be useful for identifying population groups most at risk of TB, or revealing epidemiological patterns not addressed in this analysis.

These results provide a nuanced description of TB epidemiology for a population group currently contributing over two-thirds of new TB cases in the United States. These findings support the development of TB services that could accelerate progress towards TB elimination in the United States and may also be relevant for understanding trends in other high-income countries.

## Supplementary Material

Hill_Epidemics_2020_suppl

## Figures and Tables

**Table 1:** Incidence risk ratios for 2016, relative to overall non-U.S.-born rate.

	Country	Incidence	Raw IRR	Adj. IRR (95 % CI)
	Somalia	88.2	6.83	8.86 (6.78, 11.52)
	Ethiopia	59.9	4.64	6.71 (5.16, 8.69)
Largest IRRs	Myanmar (Burma)	78.4	6.07	6.37 (4.90, 8.27)
	Liberia	38.3	2.96	5.58 (4.14, 7.50)
	Kenya	33.4	2.59	5.56 (4.20, 7.35)
	Mexico	9.7	0.75	0.64 (0.50, 0.81)
	Philippines	37.3	2.89	2.58 (2.01, 3.30)
Most cases	India	20.7	1.60	1.33 (1.04, 1.71)
	Vietnam	34.8	2.70	3.15 (2.45, 4.05)
	China	15.1	1.17	0.70 (0.54, 0.89)
	United Kingdom	0.7	0.05	0.03 (0.02, 0.05)
	Germany	0.2	0.01	0.03 (0.02, 0.05)
Smallest IRRs	Netherlands	0.0	0.00	0.03 (0.01, 0.08)
	Australia	0.0	0.00	0.02 (0.01, 0.05)
	Canada	0.6	0.05	0.02 (0.01, 0.03)

Incidence refers to annual incidence rate per 100,000 population; IRR = incidence risk ratio relative to overall non-U.S.-born rate in 2016 of 12.9 per 100,000; Adj. = adjusted; CI = confidence interval. Countries in ‘Most cases’ are in decreasing order of number of cases.
