# Supplementary material for: High-resolution estimates of tuberculosis incidence among non-U.S.-born persons residing in the United States, 2000–2016
Source: Epidemics. Author manuscript; Available in PMC 2021 Dec 1. (PMC7808561; doi:10.1016/j.epidem.2020.100419)
Supplement: Hill_Epidemics_2020_suppl [file NIHMS1658878-supplement-Hill_Epidemics_2020_suppl.docx]

Supplementary Table: Incidence risk ratios for 2016 among top 98 countries by average U.S.-population 2000-2016, relative to overall non-U.S.-born

| Country | ISO3 | Incidence | Raw IRR | Adj. IRR (95% CI) |
| --- | --- | --- | --- | --- |
| Somalia | SOM | 88.2 | 6.83 | 8.86 (6.78, 11.52) |
| Ethiopia | ETH | 59.9 | 4.64 | 6.71 (5.16, 8.69) |
| Myanmar (Burma) | MMR | 78.4 | 6.07 | 6.37 (4.90, 8.27) |
| Liberia | LBR | 38.3 | 2.96 | 5.58 (4.14, 7.50) |
| Kenya | KEN | 33.4 | 2.59 | 5.56 (4.20, 7.35) |
| Sudan | SDN | 70.2 | 5.43 | 5.20 (3.72, 7.32) |
| Nepal | NPL | 70.8 | 5.48 | 4.79 (3.63, 6.30) |
| Sierra Leone | SLE | 30.0 | 2.32 | 4.69 (3.31, 6.57) |
| Indonesia | IDN | 38.3 | 2.96 | 3.82 (2.87, 5.06) |
| Cameroon | CMR | 27.8 | 2.15 | 3.65 (2.68, 5.00) |
| Vietnam | VNM | 34.8 | 2.70 | 3.15 (2.45, 4.05) |
| Cambodia | KHM | 37.8 | 2.93 | 2.84 (2.13, 3.74) |
| Philippines | PHL | 37.3 | 2.89 | 2.58 (2.01, 3.30) |
| Laos | LAO | 36.3 | 2.81 | 2.41 (1.80, 3.23) |
| Haiti | HTI | 23.4 | 1.81 | 2.40 (1.87, 3.10) |
| Guatemala | GTM | 18.0 | 1.40 | 2.24 (1.74, 2.89) |
| Honduras | HND | 20.7 | 1.60 | 2.24 (1.72, 2.90) |
| Ecuador | ECU | 13.8 | 1.07 | 2.07 (1.58, 2.69) |
| Peru | PER | 13.7 | 1.06 | 1.96 (1.52, 2.54) |
| Bangladesh | BGD | 21.8 | 1.69 | 1.77 (1.36, 2.34) |
| Nigeria | NGA | 27.2 | 2.11 | 1.74 (1.32, 2.28) |
| Afghanistan | AFG | 26.5 | 2.05 | 1.61 (1.16, 2.22) |
| Bolivia | BOL | 15.9 | 1.23 | 1.51 (1.09, 2.06) |
| Thailand | THA | 15.8 | 1.22 | 1.40 (1.07, 1.85) |
| India | IND | 20.7 | 1.60 | 1.33 (1.04, 1.71) |
| Pakistan | PAK | 20.5 | 1.58 | 1.22 (0.93, 1.61) |
| Morocco | MAR | 8.3 | 0.64 | 1.20 (0.83, 1.73) |
| Malaysia | MYS | 11.8 | 0.91 | 1.14 (0.76, 1.70) |
| South Africa | ZAF | 6.4 | 0.50 | 1.14 (0.80, 1.61) |
| Cape Verde | CPV | 8.6 | 0.67 | 1.11 (0.73, 1.71) |
| Bosnia & Herzegovina | BIH | 14.4 | 1.12 | 1.05 (0.69, 1.56) |
| Yemen | YEM | 23.3 | 1.80 | 0.90 (0.61, 1.32) |
| Ghana | GHA | 13.1 | 1.02 | 0.86 (0.61, 1.18) |
| El Salvador | SLV | 7.8 | 0.61 | 0.71 (0.54, 0.92) |
| China | CHN | 15.1 | 1.17 | 0.70 (0.54, 0.89) |
| Mexico | MEX | 9.7 | 0.75 | 0.64 (0.50, 0.81) |
| Guyana | GUY | 6.6 | 0.51 | 0.63 (0.45, 0.87) |
| Belize | BLZ | 7.7 | 0.60 | 0.58 (0.35, 1.00) |
| Dominican Republic | DOM | 5.2 | 0.41 | 0.55 (0.42, 0.72) |
| Nicaragua | NIC | 6.5 | 0.51 | 0.53 (0.38, 0.75) |
| Uzbekistan | UZB | 7.2 | 0.56 | 0.49 (0.30, 0.82) |
| Panama | PAN | 4.8 | 0.37 | 0.46 (0.28, 0.73) |
| Croatia | HRV | 0.0 | 0.00 | 0.45 (0.25, 0.80) |
| Hong Kong SAR China | HKG | 11.8 | 0.91 | 0.42 (0.30, 0.58) |
| Portugal | PRT | 0.5 | 0.04 | 0.42 (0.27, 0.65) |
| Sri Lanka | LKA | 3.5 | 0.27 | 0.41 (0.26, 0.67) |
| Moldova | MDA | 7.0 | 0.54 | 0.40 (0.23, 0.71) |
| Fiji | FJI | 4.7 | 0.36 | 0.40 (0.22, 0.71) |
| Romania | ROU | 4.5 | 0.35 | 0.39 (0.28, 0.55) |
| South Korea | KOR | 8.0 | 0.62 | 0.36 (0.28, 0.47) |
| Iraq | IRQ | 4.1 | 0.32 | 0.35 (0.24, 0.50) |
| Saudi Arabia | SAU | 6.0 | 0.47 | 0.34 (0.23, 0.51) |
| Ukraine | UKR | 6.1 | 0.47 | 0.33 (0.24, 0.46) |
| Russia | RUS | 4.7 | 0.36 | 0.33 (0.25, 0.45) |
| Armenia | ARM | 2.1 | 0.16 | 0.28 (0.18, 0.45) |
| Colombia | COL | 6.4 | 0.50 | 0.28 (0.21, 0.37) |
| Albania | ALB | 5.6 | 0.44 | 0.28 (0.18, 0.43) |
| Lithuania | LTU | 0.0 | 0.00 | 0.27 (0.15, 0.50) |
| Turkey | TUR | 4.6 | 0.35 | 0.26 (0.18, 0.39) |
| Uruguay | URY | 3.8 | 0.30 | 0.25 (0.14, 0.46) |
| Brazil | BRA | 3.6 | 0.28 | 0.24 (0.18, 0.33) |
| Trinidad & Tobago | TTO | 2.9 | 0.23 | 0.22 (0.15, 0.33) |
| Egypt | EGY | 1.9 | 0.15 | 0.20 (0.13, 0.30) |
| Syria | SYR | 4.0 | 0.31 | 0.19 (0.11, 0.34) |
| Argentina | ARG | 5.1 | 0.40 | 0.18 (0.12, 0.26) |
| Jordan | JOR | 2.2 | 0.17 | 0.16 (0.09, 0.30) |
| Poland | POL | 1.4 | 0.11 | 0.16 (0.11, 0.22) |
| Iran | IRN | 2.1 | 0.16 | 0.16 (0.11, 0.22) |
| Greece | GRC | 2.9 | 0.23 | 0.15 (0.08, 0.28) |
| Bulgaria | BGR | 5.1 | 0.40 | 0.15 (0.09, 0.27) |
| Taiwan | TWN | 7.9 | 0.61 | 0.14 (0.11, 0.20) |
| Venezuela | VEN | 2.9 | 0.22 | 0.14 (0.10, 0.21) |
| Grenada | GRD | 2.8 | 0.22 | 0.14 (0.06, 0.33) |
| Belgium | BEL | 0.0 | 0.00 | 0.11 (0.04, 0.28) |
| Spain | ESP | 3.1 | 0.24 | 0.11 (0.07, 0.18) |
| Costa Rica | CRI | 1.1 | 0.08 | 0.11 (0.06, 0.19) |
| Cuba | CUB | 1.9 | 0.14 | 0.11 (0.08, 0.14) |
| Jamaica | JAM | 1.4 | 0.11 | 0.10 (0.07, 0.15) |
| Italy | ITA | 3.0 | 0.23 | 0.10 (0.06, 0.16) |
| Barbados | BRB | 0.0 | 0.00 | 0.10 (0.04, 0.26) |
| Austria | AUT | 0.0 | 0.00 | 0.09 (0.04, 0.24) |
| Belarus | BLR | 0.0 | 0.00 | 0.09 (0.04, 0.19) |
| Hungary | HUN | 2.7 | 0.21 | 0.08 (0.04, 0.17) |
| Chile | CHL | 0.0 | 0.00 | 0.08 (0.04, 0.14) |
| Denmark | DNK | 0.0 | 0.00 | 0.08 (0.03, 0.19) |
| Lebanon | LBN | 1.5 | 0.12 | 0.07 (0.04, 0.14) |
| France | FRA | 2.3 | 0.18 | 0.07 (0.05, 0.11) |
| Czechia | CZE | 2.0 | 0.16 | 0.06 (0.03, 0.16) |
| Japan | JPN | 2.0 | 0.16 | 0.06 (0.04, 0.08) |
| Israel | ISR | 1.3 | 0.10 | 0.05 (0.02, 0.09) |
| Ireland | IRL | 1.7 | 0.13 | 0.04 (0.02, 0.08) |
| Switzerland | CHE | 0.0 | 0.00 | 0.04 (0.01, 0.11) |
| Sweden | SWE | 0.0 | 0.00 | 0.04 (0.01, 0.10) |
| United Kingdom | GBR | 0.7 | 0.05 | 0.03 (0.02, 0.05) |
| Germany | DEU | 0.2 | 0.01 | 0.03 (0.02, 0.05) |
| Netherlands | NLD | 0.0 | 0.00 | 0.03 (0.01, 0.08) |
| Australia | AUS | 0.0 | 0.00 | 0.02 (0.01, 0.05) |
| Canada | CAN | 0.6 | 0.05 | 0.02 (0.01, 0.03) |

ISO3 = ISO 3166-1 alpha-3 country code; Incidence refers to annual incidence rate per 100,000 population; IRR = incidence risk ratio; Adj. = adjusted; CI = confidence interval
